# Association between diabetes and pesticides: a case-control study among Thai farmers

**DOI:** 10.1186/s12199-018-0692-5

**Published:** 2018-01-27

**Authors:** Chudchawal Juntarawijit, Yuwayong Juntarawijit

**Affiliations:** 1 Department of Natural Resources and Environment, Faculty of Agriculture, Natural Resources and Environment, Naresuan University, Muang District, Phitsanulok, 65000 Thailand; 2 Faculty of Nursing, Naresuan University, Phitsanulok, Thailand

**Keywords:** Diabetes, Pesticides, Environmental exposure, Insecticides, Herbicides

## Abstract

**Background:**

Pesticides are an agricultural chemical suspected to be a significant contributor to a global diabetes pandemic. The purpose of this study was to confirm previous findings of the link between diabetes and some agricultural pesticides and to identify the particular pesticides that are most likely to pose a risk of diabetes in the community.

**Methods:**

A population-based case-controlled study was conducted among residents in the Bang Rakam district of Phitsanulok Province in Thailand. Lifetime pesticide exposure and other relevant data were collected from 866 participating cases with diabetes mellitus and 1021 healthy controls.

**Results:**

After adjusting for gender, age, BMI, cigarette smoking, alcohol consumption, family history of diabetes, and occupation, it was found that the prevalence of diabetes was positively associated with exposure to all types of pesticides, including insecticides, herbicides, fungicides, rodenticides, and molluscicides, with exposure to rodenticides being statistically significant (OR = 1.35; 95%CI 1.04–1.76). Among 35 individual brand-named pesticides investigated, we found statistically significant ORs with three insecticides, including one organochlorine [endosulfan (OR = 1.40; 95%CI 1.01–1.95)], one organophosphate [mevinphos (OR = 2.22; 95%CI 1.17–4.19)], and one carbamate [carbaryl/Sevin (OR = 1.50; 95%CI 1.02–2.19)]; and one fungicides [benlate (OR = 2.08; 95%CI 1.03–4.20)].

**Conclusions:**

Our results suggest that the occurrence of diabetes among Thai farmer was associated with pesticide exposure. This finding is in line with previous epidemiological and animal studies. Further study using a larger sample size is needed to confirm the relationship and to identify the more toxic compounds.

## Background

Diabetes is an important public health problem with 415 million adults (aged 20–79 years) worldwide suffering the disease in 2015. By 2040, the figure will rise to 642 million [1]. In Thailand, diabetes among adult aged ≥ 18 years drastically increased from 6.9% in 2009 to 8.9% (4.8 million people) in 2014 [2]. The confirmed causal factors were gender, genetic, and overweight [3]. In addition, the disease was also strongly associated with many lifestyle behaviors, including lack of active exercise [4], cigarette smoking [5] and alcohol consumption [3], sleeping patterns [6, 7], and pregnancy status [8]. Recent research has also suggested environmental contamination by agricultural chemicals to be a causal factor [9]. The suspected compounds are arsenic, mercury, phthalates, bisphenol A (BPA), and persistent organic pollutants (POPs), especially PCBs, dioxin, and pesticides [10–12].

Pesticides have received greater attention as they are the most commonly compounds in use today, with more than 2.3 billion kilograms used worldwide in 2001 [13]. In an Agricultural Health Study (AHS) project in the USA, a large cohort study of 33,457 pesticide applicators reported an association between diabetes and seven organochlorine pesticides, including aldrin, chlordane, heptachlor, dichlorvos, trichlorfon, alachlor, and cyanazine [14]. Further study of this program using data of the wives of 13,637 pesticide applicators identified more diabetic risk compounds, including three organophosphates, including fonofos (OR = 1.56; 95%CI 1.11–2.19), phorate (OR = 1.57; 95%CI 1.14–2.16), and parathion (OR = 1.61; 95%CI 1.05–2.46); one organochlorine, dieldrin (OR = 1.99; 95%CI 1.12–3.54); and one herbicide, 2,4,5-T/2,4,5-TP (OR = 1.59; 95%CI 1.00–2.51) [15]. Study among women who were involved in mixing or applying pesticides, or fixing pesticide appliances, also found diabetes and pesticide exposure with odds ratios of 2.2 (95%CI 1.5–3.3) [16]. From these studies, it is evident that gender plays a significant role, as well as the several other factors which influence diabetic occurrence, including race, ethnicity and economic status, education level, and nutritional conditions [17]. A study among Mexican-American farmers found an association with several organochlorine pesticides, including *trans*-nonachlor, oxychlordane, β-hexachlorocyclohexane, *p,p*′-DDT, and *p,p*′-DDE [18]. Another study using data from Native Americans found PCBs, dichlorodiphenyldichloroethylene (DDE), and hexachlorobenzene (HCB) to be associated with diabetes [19].

With currently limited information, several mechanisms have been suggested to explain the linkage between environmental compounds and diabetes. In animal and cell studies, it was found that organochlorine pesticide and some other POPs reduce cell metabolic functions and cause obesity [20]. There has been evidence that pollutant compounds affect insulin secretion, insulin action, and glucose homeostasis [21–23], and elsewhere, physiological and oxidative stress, and cholinesterase inhibition [24]. Recently, Park et al. [25] proposed a molecular scheme to explain how these chemicals caused metabolic disorders by damaging the mitochondria, which are organelles that act as powerhouses of the cells.

Information on diabetes and pesticide linkage is relatively new, and a significant data gap still remains [12]. This purpose of this case-control study was to identify more specific compounds which are, or may be, associated with diabetes, using data on Thai rural residents. With the differences in the health backgrounds, life styles, and agricultural practices and behaviors of this particular group, the results can be compared to those previously reported in the literature which has mostly been conducted in the Western world.

## Methods

This study was a population-based case-control study. It was conducted from February to May 2016 in Bang Rakam district, located in Phitsanulok Province, 377 km north of Bangkok, Thailand. The district has a population of 94,980, most of whom are rice farmers [26].

### Study subjects

The participants were medical diagnosed diabetic patients from seven local, sub-district hospitals in Bang Rakam district. The 7 hospitals were randomly selected from the 21 sub-district hospitals in the target area. From the 2832 patients who sought out-patient follow-up service from the selected 7 hospitals, 1000 cases (approximately 35%) were randomly selected in proportional manner from the hospitals for initial interview. The controls were close neighbors who were without diabetes, having the same gender and aged within ± 5 years of the cases’ age. Due to missing information on key data for some of the initial 2100 patients, such as pesticides usage, age, weight, and behavior, the final data analysis was conducted among 1887 subjects (866 cases and 1021 controls).

This study was approved by the Ethics Board of Naresuan University (project number 402/59). Written informed consent for an interview was obtained from each subject.

### Questionnaire

Data on pesticide exposure was collected using a structured questionnaire. Questions were derived from the Agricultural Health Study Questionnaire (https://aghealth.nih.gov/collaboration/questionnaires.html). Information collected included demographic data, occupation, health behaviors, body weight, and height. Body mass index (BMI) was calculated as weight (kg) divided by height squared (m^2^). Height and weight of participants were measured by interviewers. For eating behavior, amount and frequency of eating vegetable, fruit, and sweet were collected. The amount of food consumption was categorized to less than a cup/glass, about a cup/glass, and more than a cup/glass. Data on eating frequency was categorized to never eating or eat less than once a month, once a month, 2–3 times per month, once a week, twice a week, 3–4 times per week, 5–6 times per week, once a day, and twice a day or more. A consumption rate per week (cup/glass per week) was then calculated, and the quartile of this rate was used for prediction of diabetes risk. Data on lifestyle (morningness-eveningness) was also collected. It is a self-reported behavior. Morning types (larks) referred to those who are usually active during the day and wake up early in the morning. Evening types (owls) are those who feel alert in the night and wake up late in the morning.

Lifetime pesticide use and use type, mixing or applying, were analyzed. Pesticides were categorized into five groups: insecticides (organochlorine, organophosphate, carbamate, and pyrethoid), fungicides, herbicides, rodenticides, and molluscicides. For each pesticide, data collected were numbers of years using pesticides (< 1, 2–5, 6–10, 11–20, and > 20 years) and numbers of days in each year using pesticides (< 5, 5–9, 10–19, 20–39, 40–59, 60–150, and > 150 days). Cumulative days of exposure were calculated from duration by frequency data using the midpoints of the reported categories. For each group of pesticides, the exposure duration was divided into quartiles of cumulative days of exposure. Among all types of pesticides, exposure data of 35 specific pesticides was collected.

The study subjects were interviewed by 50 village health volunteers, 5–10 in each sub-district. Prior to data collection, all the health volunteers were trained on how to interview and use the questionnaire.

### Statistical analysis

Collected data was analyzed using IBM SPSS Statistics (Version 19) and OpenEpi (Version 3.01). *P* values < 0.05 were considered statistically significant. In the association between diabetes and pesticides exposure, odds ratio (OR) was calculated for each group of pesticides (insecticides, herbicides, fungicides, rodenticides, and molluscicides) and 35 individual compounds. Both crude and adjusted ORs with 95% confidence intervals (CIs) were presented. Adjusted ORs were analyzed using multiple logistic regression controlled for gender, age (continuous), BMI (continuous), cigarette smoking (packs/year), alcohol consumption (glass/week), family history of diabetes (yes, no), and occupation (farmer, non-farmer, other). The control variables were those with difference between cases and controls in addition to the fundamental confounding factors.

For exposure duration to a group of pesticides, cumulative exposure days were computed and categorized into quartile of exposure days. The diabetic risk was then predicted, using quartile 1 as a reference. For each specific pesticide, exposure was categorized as ever vs. never used. The cumulative exposure days were not performed because the number of subject reported using each individual pesticide was too small.

## Results

Both case and control groups had a similar demographic data with a comparable proportion of men, 35% for control and 33% for case (*P* = 0.36) (Table 1). Comparing case to control, the mean age (57.68 ± 9.00 vs. 57.64 ± 9.17) and age in each categories did not differ significantly (*P* = 0.99). However, case had a higher average BMI (25.67 ± 6.99) than control (24.06 ± 6.04). Most of the study subjects are farmer, 78.9% for case and 75.3% for control. An average number of alcohol consumption of case was 7.16 ± 2.34 glass per year, significantly higher than that of control (6.85 ± 2.60) (*P* < 0.01). However, case smoked cigarette in less amount (1.41 ± 5.80 vs 2.56 ± 9.36 pack/year, case to control) (*P* < 0.01). The two groups of subjects had no difference in lifestyle (morningness-eveningness), ever exercise during the past 12 months, and frequency of TV watching during leisure time; and an amount of vegetable, fruit, and sweet intake.

**Table 1:** General characteristics and variables between case and control groups

Characteristic	Case (*n* = 866)*	Control (*n* = 1021)	*P* value**
Men (%)	286 (33.0)	357 (35.0)	0.38
Age (years)			0.99
≤ 44	57 (6.6)	71 (7.0)	
45–54	246 (28.4)	293 (28.7)	
55–64	373 (43.1)	435 (42.6)	
65–74	162 (18.7)	186 (18.2)	
≥ 75	28 (3.2)	36 (3.5)	
Mean age ± s.d.	57.68 ± 9.00	57.64 ± 9.17	0.92
Median (min-max)	58.0 (25–84)	58.0 (15–86)	
BMI (kg/m^2^)			< 0.01
< 18.50	27 (3.1)	66 (6.5)	
18.50–22.90	227 (26.2)	379 (37.1)	
23.00–24.90	178 (20.6)	182 (17.8)	
25.00–29.90	283 (32.7)	279 (27.3)	
> 30.00	115 (13.3)	76 (7.4)	
Missing	36 (4.2)	39 (3.8)	
Mean ± s.d.	25.70 ± 6.94	24.06 ± 6.04	< 0.01
Median (min-max)	24.8 (13.8–141.7)	23.5 (13.8–166.7)	
Occupation			0.02
Farmer	650 (75.1)	805 (78.8)	
Non-farming private business	164 (18.9)	145 (14.2)	
Other	49 (5.7)	70 (6.9)	
Missing	3 (0.3)	1 (0.1)	
Cigarette smoke			< 0.01
Never smoked cigarettes	719 (83.0)	798 (78.2)	
Have smoked cigarettes	62 (7.2)	67 (6.6)	
Current smoker	77 (8.9)	144 (14.1)	
Missing	8 (0.9)	12 (1.2)	
Mean pack/year ± s.d.	1.41 ± 5.80	2.56 ± 9.36	< 0.01
Median pack/year (min-max)	0.0 (0.0–75.0)	0.0 (0.0–120)	
Alcohol consumption			< 0.01
Never drank	616 (71.1)	647 (63.4)	
Former drinker	112 (12.9)	112 (11.0)	
Current drinker	128 (14.8)	244 (23.9)	
Missing	10 (1.2)	18 (1.8)	
Mean glasses per week ± s.d.	3.11 ± 11.6	4.8 ± 14.4	< 0.01
Median glasses per week (min-max)	0.0 (0.0–84.0)	0.0 (0.0–84.0)	
Having family history of diabetes			< 0.01
Yes	500 (57.7)	365 (35.7)	
No	355 (41.0)	643 (63.0)	
Missing	11 (1.3)	13 (1.3)	
Hours of sleep per day			0.95
4–6	77 (8.9)	90 (8.8)	
7–9	692 (79.9)	826 (80.9)	
10–12	54 (6.2)	68 (6.7)	
Missing	43 (5.0)	37 (3.6)	
Mean ± s.d.	7.92 ± 1.12	7.93 ± 1.13	0.85
Lifestyle (morningness-eveningness)***			0.77
Morning types (larks)	799 (92.3)	948 (92.9)	
Evening types (owls)	53 (6.1)	67 (6.6)	
Missing	14 (1.6)	6 (0.6)	
Ever exercise during the past 12 months			0.60
Yes	319 (36.8)	387 (37.9)	
No	535 (61.8)	617 (60.4)	
Missing	12 (1.4)	17 (1.7)	
Watching TV during leisure time			0.43
Never	34 (3.9)	37 (3.6)	
Rarely	147 (17.0)	155 (15.2)	
Sometimes	331 (38.2)	368 (36.0)	
Very often	254 (29.3)	337 (33.0)	
Always	54 (6.2)	58 (5.7)	
Missing	46 (5.3)	66 (6.5)	
Intakes of vegetables (cups per week)			0.72
Quartile1	126 (14.5)	164 (16.1)	
Quartile2	315 (36.4)	358 (35.1)	
Quartile3	197 (22.7)	238 (23.3)	
Quartile4	177 (20.4)	196 (19.2)	
Missing	51 (5.9)	65 (6.4)	
Mean cups per week ± s.d.	12.40 ± 11.58	12.00 ± 11.21	0.46
Intakes of fruits (cups per week)			0.95
Quartile1	254 (29.3)	296 (29.0)	
Quartile2	263 (30.4)	314 (30.8)	
Quartile3	148 (17.1)	163 (16.0)	
Quartile4	149 (17.2)	178 (17.4)	
Missing	52 (6.0)	70 (6.9)	
Mean cups per week ± s.d.	5.44 ± 6.91	5.29 ± 6.76	0.64
Intakes of sweets (cups per week)			0.22
Quartile1	65 (7.5)	67 (6.6)	
Quartile2	316 (36.5)	352 (34.5)	
Quartile3	159 (18.4)	223 (21.8)	
Quartile4	280 (32.3)	311 (30.5)	
Missing	46 (5.3)	68 (6.7)	
Mean cups per week ± s.d.	2.91 ± 4.56	2.98 ± 4.57	0.75

*N was 1021 for control and 866 for case unless otherwise indicated

**χ^2^ test for categorical data; *t* test for continuous data

***Morning types (larks) were those who wake up early and active during the day; evening types (owls) were usually feel alert in the night and wake up late in the morning

Association between different types of pesticide and diabetes were presented in Table 2. It was found that ever use of any pesticides cannot predict risk of diabetes. Experience using any types of pesticides was positively associated with diabetes but the association were statistically significant only for rodenticide (adjusted OR = 1.35; 95%CI 1.04–1.76). For insecticides, year of exposure to insecticides and cumulative days failed to predict risk of diabetes. For fungicides, diabetic risk seems to correlate with both year of exposure and exposure days, but none with statistical significance. For herbicides and molluscicides, both year of exposure and exposure days were inversely associated with the disease. For rodenticides, only exposure days was positively correlated with diabetes.

**Table 2:** Associations between different types of pesticides and diabetes

	Case		Control		OR (95%CI)	Adjusted OR* (95%CI)
	Exposed *n* %		Exposed n %			
Pesticides (any) (*N* = 1867)	468	54.5	556	55.1	0.98 (0.81–1.17)	0.94 (0.75–1.17)
Insecticides (any) (*N* = 1876)	433	50.2	500	49.4	1.03 (0.86–1.24)	1.04 (0.84–1.29)
Number of years using the insecticides (*N* = 933)						
1–5	50	11.6	53	10.6	Referent	Referent
6–10	82	18.9	102	20.4	0.85 (0.53–1.38)	0.83 (0.49–1.41)
11–20	99	22.9	126	25.2	0.83 (0.52–1.33)	0.67 (0.40–1.12)
21–30	105	24.3	110	22.0	1.01 (0.63–1.62)	1.01 (0.61–1.68)
> 30	97	22.4	109	21.8	0.94 (0.59–1.51)	0.80 (0.47–1.34)
*P* for trend***					0.78	0.80
Number of days using insecticides (quartile) (*N* = 920)						
Quartile1	119	27.5	128	25.6	Referent	Referent
Quartile2	99	22.9	116	23.2	0.92 (0.64–1.32)	1.02 (0.68–1.51)
Quartile3	113	26.1	130	26.0	0.94 (0.66–1.33)	0.83 (0.56–1.23)
Quartile4	97	22.4	118	23.6	0.88 (0.61–1.28)	0.86 (0.58–1.28)
*P* for trend					0.12	0.18
Fungicides (any) (*N* = 1881)	336	38.9	387	38.0	1.04 (0.86–1.25)	1.00 (0.80–1.24)
Number of years using the fungicides (*N* = 723)						
1–5	46	13.7	55	14.2	Referent	Referent
6–10	65	19.4	88	22.7	0.88 (0.53–1.47)	0.87 (0.50–1.51)
11–20	83	24.7	105	27.1	0.95 (0.58–1.54)	0.93 (0.55–1.58)
21–30	80	23.8	69	17.8	1.39 (0.84–2.30)	1.55 (0.90–2.69)
> 30	62	18.5	70	18.1	1.06 (0.63–1.78)	1.03 (0.58–1.82)
*P* for trend					0.37	0.46
Number of days using fungicides (quartile) (*N* = 672)						
Quartile1	96	28.6	117	30.2	Referent	Referent
Quartile2	83	24.7	93	24.0	1.09 (0.73–1.62)	1.06 (0.69–1.64)
Quartile3	72	21.4	70	18.1	1.25 (0.82–1.92)	1.13 (0.70–1.80)
Quartile4	62	18.5	79	20.4	0.96 (0.62–1.47)	1.02 (0.64–1.62)
*P* for trend					0.96	0.71
Herbicides (any) (*N* = 1882)	418	48.4	491	48.2	1.01 (0.84–1.21)	1.13 (0.76–1.67)
Number of years using the herbicides (*N* = 909)						
1–5	55	13.2	56	11.4	Referent	Referent
6–10	83	19.9	118	24.0	0.72 (0.45–1.14)	0.60 (0.36–0.99)**
11–20	102	24.4	113	23.0	0.92 (0.58–1.45)	0.74 (0.45–1.22)
21–30	99	23.7	104	21.2	0.97 (0.61–1.54)	0.84 (0.51–1.38)
> 30	79	18.9	100	20.4	0.80(0.50–1.29)	0.63(0.37–1.06)
*P* for trend					0.78	0.48
Number of days using herbicides (quartile) (*N* = 844)						
Quartile1	124	29.7	142	28.9	Referent	Referent
Quartile2	76	18.2	87	17.7	1.00 (0.68–1.48)	0.90 (0.59–1.37)
Quartile3	96	23.0	109	22.2	0.99 (0.69–1.43)	0.93 (0.62–1.38)
Quartile4	92	22.0	118	24.0	0.91 (0.64–1.30)	0.84 (0.57–1.26)
*P* for trend					0.17	0.13
Rodenticides (any) (*N* = 1886)	227	26.3	223	21.8	1.28 (1.04–1.58)	1.35 (1.04–1.76)**
Number of years using the rodenticides (*N* = 450)						
1–5	79	34.8	64	28.7	Referent	Referent
6–10	60	26.4	63	28.3	0.77 (0.48–1.25)	0.81 (0.48–1.37)
11–20	45	19.8	43	19.3	0.85 (0.50–1.44)	1.03 (0.57–1.84)
21–30	19	8.4	24	10.8	0.64 (0.32–1.27)	0.87 (0.40–1.88)
> 30	24	10.6	29	13.0	0.67 (0.36–1.26)	0.83 (0.42–1.66)
*P* for trend					0.08	0.44
Number of days using rodenticides (quartile) (*N* = 425)						
Quartile1	65	28.6	53	23.8	Referent	Referent
Quartile2	68	30.0	56	25.1	0.99 (0.60–1.64)	1.05 (0.61–1.83)
Quartile3	37	16.3	52	23.3	0.58 (0.33–1.01)	0.75 (0.41–1.38)
Quartile4	46	20.3	48	22.0	0.78 (0.45–1.35)	1.09 (0.60–1.99)
*P* for trend					0.31	0.98
Molluscicides (any) (*N* = 1879)	243	28.2	255	25.1	1.17 (0.95–1.44)	1.26 (0.96–1.64)
Number of years using the molluscicides (*N* = 498)						
1–5	76	31.3	71	27.8	Referent	Referent
6–10	70	28.8	74	29.0	0.88 (0.56–1.40)	0.91 (0.55–1.51)
11–20	50	20.6	63	24.7	0.74 (0.45–1.21)	0.81 (0.47–1.39)
21–30	23	9.0	26	10.2	0.83 (0.43–1.58)	0.88 (0.43–1.80)
> 30	24	9.9	21	8.2	1.07 (0.55–2.09)	1.20 (0.58–2.48)
*P* for trend					0.70	0.39
Number of days using molluscicides (quartile) (*N* = 471)						
Quartile1	94	38.7	80	31.4	Referent	Referent
Quartile2	37	15.2	34	13.3	0.93 (0.53–1.61)	1.00 (0.54–1.83)
Quartile3	62	25.5	72	28.2	0.73 (0.47–1.15)	0.76 (0.47–1.25)
Quartile4	38	15.6	54	21.2	0.60 (0.36–1.00)**	0.70 (0.40–1.23)
*P* for trend					0.01**	0.07

*Adjusted for gender, age (continuous), BMI (continuous), cigarette smoking (packs/year), alcohol consumption (glasses/week), family history of diabetes (yes, no), and occupation (farmer, non-farmer, other)

**Statistically significant (*P* < 0.05)

***P values for linear trend were derived using a continuous variable with the midpoint value of each category

Table 3 presents the ORs of each individual pesticide for diabetes. Among 20 compound investigated, 15 were positively associated with diabetes, 3 with statistical significance [endosulfan (OR = 1.40; 95%CI 1.01–1.95), mevinphos (OR = 2.22; 95%CI 1.17–4.19), and carbaryl/Sevin (OR = 1.50; 95%CI 1.02–2.19)]. For herbicides, of the 6 compounds investigated, 4 showed increased ORs. Study also found 7 out of 9 fungicides to be correlated with diabetes, 1 with statistical significant [benlate (OR = 2.08; 95%CI 1.03–4.20).

**Table 3:** Associations between individual pesticide and diabetes

Exposed		Case *n* (%)	Control *n* (%)	OR (95%CI)	Adjusted OR* (95%CI)
Organochlorine insecticides					
Aldrin	Yes	7 (1.5)	11 (2.0)	0.77 (0.30–1.99)	0.79 (0.28–2.23)
	No	445 (98.5)	536 (98.0)		
Chlordane	Yes	12 (2.7)	12 (2.2)	1.22 (0.54–2.75)	1.35 (0.56–3.24)
	No	439 (97.3)	536 (97.8)		
Dieldrin	Yes	6 (1.3)	11 (2.0)	0.65 (0.24–1.78)	0.67 (0.22–2.02)
	No	447 (98.7)	536 (98.0)		
DDT	Yes	32 (7.1)	48 (8.8)	0.79 (0.50–1.26)	0.83 (0.50–1.37)
	No	420 (92.9)	500 (91.2)		
Endosulfan	Yes	112 (24.6)	105 (19.1)	1.38 (1.02–1.87)**	1.40 (1.01–1.95)**
	No	343 (75.4)	444 (80.9)		
Heptachlor	Yes	9 (2.0)	13 (2.4)	0.83 (0.35–1.97)	1.13 (0.46–2.80)
	No	444 (98.0)	535 (97.6)		
Organophosphate insecticides					
Chlorpyrifos	Yes	76 (16.8)	78 (14.3)	1.21 (0.86–1.71)	1.20 (0.83–1.75)
	No	376 (83.2)	468 (85.7)		
Dicrotophos	Yes	18 (4.0)	13 (2.4)	1.70 (0.82–3.51)	2.12 (0.97–4.61)
	No	436 (96.0)	535 (97.6)		
Dichlorvos	Yes	10 (2.2)	12 (2.2)	1.01 (0.43–2.36)	1.03 (0.41–2.62)
	No	442 (97.8)	536 (97.8)		
EPN (ethyl-p-nitrophenyl)	Yes	50 (11.0)	52 (9.5)	1.19 (0.79–1.79)	1.29 (0.83–2.02)
	No	403 (89.0)	497 (90.5)		
Folidol/parathion	Yes	105 (23.2)	135 (24.5)	0.93 (0.70–1.25)	0.83 (0.60–1.15)
	No	348 (76.8)	417 (75.5)		
Methamidophos/Tamaron	Yes	43 (9.5)	49 (9.0)	1.07 (0.69–1.64)	1.04 (0.65–1.65)
	No	409 (90.5)	497 (91.0)		
Mevinphos	Yes	26 (5.7)	20 (3.6)	1.61 (0.89–2.92)	2.22 (1.17–4.19)**
	No	427 (94.3)	528 (96.4)		
Monocrotophos	Yes	26 (5.8)	26 (4.7)	1.23 (0.70–2.15)	1.29 (0.72–2.32)
	No	426 (94.2)	523 (95.3)		
Carbamate insecticides					
Carbaryl/Sevin	Yes	81 (17.8)	70 (12.8)	1.48 (1.05–2.10)**	1.50 (1.02–2.19)**
	No	373 (82.2)	478 (87.2)		
Carbofuran/Furadan	Yes	112 (24.7)	129 (23.5)	1.07 (0.80–1.43)	1.23 (0.90–1.70)
	No	342 (75.3)	421 (76.5)		
Carbosulfan	Yes	54 (11.9)	50 (9.1)	1.35 (0.90–2.03)	1.44 (0.94–1.12)
	No	398 (88.1)	498 (90.9)		
Methomyl	Yes	89 (19.6)	85 (15.5)	1.33 (0.96–1.84)	1.23 (0.86–1.76)
	No	365 (80.4)	462 (84.5)		
Pyrethoid insecticides					
Abamectin	Yes	220 (48.6)	290 (52.4)	0.86 (0.67–1.10)	0.79 (0.60–1.04)
	No	233 (51.4)	263 (47.6)		
Permethrin/Ambush	Yes	15 (3.3)	15 (2.7)	1.23 (0.59–2.53)	1.39 (0.64–3.04)
	No	435 (96.7)	533 (97.3)		
Herbicides					
2,4-D	Yes	147 (32.1)	167 (30.1)	1.10 (0.84–1.44)	1.04 (0.78–1.39)
	No	311 (67.9)	388 (69.9)		
Alachlor	Yes	44 (9.7)	57 (10.4)	0.93 (0.61–1.40)	0.94 (0.59–1.47)
	No	409 (90.3)	490 (89.6)		
Butachlor	Yes	115 (25.1)	130 (23.7)	1.08 (0.81–1.44)	1.14 (0.83–1.56)
	No	344 (74.9)	418 (76.3)		
Glyphosate	Yes	65 (13.8)	88 (15.8)	1.17 (0.83–1.65)	1.08 (0.72–1.61)
	No	405 (86.2)	470 (84.2)		
Paraquat/Gramoxone	Yes	115 (24.8)	161 (29.0)	1.23 (0.93–1.63)	1.31 (0.97–1.79)
	No	348 (75.2)	395 (71.0)		
Propanil	Yes	94 (20.6)	129 (23.5)	0.85 (0.63–1.15)	0.81 (0.58–1.13)
	No	362 (79.4)	421 (76.5)		
Fungicides					
Benlate	Yes	24 (5.4)	16 (2.9)	1.88 (0.99–3.58)	2.08 (1.03–4.20)**
	No	424 (94.6)	531 (97.1)		
Bordeaux mixture	Yes	6 (1.3)	11 (2.0)	0.66 (0.24–1.80)	0.81 (0.29–2.29)
	No	444 (98.7)	538 (98.0)		
Carbendazim	Yes	45 (10.0)	49 (9.0)	1.13 (0.74–1.73)	1.17 (0.74–1.86)
	No	405 (90.0)	498 (91.0)		
Copper sulfate	Yes	14 (3.2)	15 (2.8)	1.15 (0.55–2.40)	1.16 (0.51–2.63)
	No	428 (96.8)	525 (97.2)		
Mancozeb	Yes	31 (6.9)	39 (7.1)	0.96 (0.59–1.57)	1.09 (0.65–1.84)
	No	421 (93.1)	510 (92.9)		
Maneb	Yes	11 (2.5)	15 (2.7)	0.89 (0.41–1.97)	1.03 (0.44–2.41)
	No	437 (97.5)	533 (97.3)		
Metalaxyl	Yes	39 (8.6)	40 (7.3)	1.20 (0.76–1.91)	1.32 (0.80–2.17)
	No	413 (91.4)	510 (92.7)		
Thiophanate-methyl	Yes	13 (2.9)	13 (2.4)	1.23 (0.56–2.68)	1.37 (0.60–3.14)
	No	435 (97.1)	534 (97.6)		
Zineb	Yes	16 (3.6)	9 (1.6)	2.22 (0.97–5.06)	2.23 (0.89–5.57)

*Adjusted for gender, age (continuous), BMI (continuous), cigarette smoking (packs/year), alcohol consumption (glasses/week), family history of diabetes (yes, no), and occupation (farmer, non-farmer, other)

**Statistically significant (*P* < 0.05)

## Discussion

In this study, the OR of diabetes was statistically associated with three types of insecticides investigated, including one organochlorine (endosulfan), one organophosphate (mevinphos), and one carbamate (carbaryl/Sevin), as well as one fungicide (benlate). The results were well supported by previous findings either from epidemiological studies or an animal study. A recent study also suggested that pesticides could affect the pancreases and interfere with insulin secretion [27] by damaging the mitochondria cells [27].

Of the six organochlorine pesticides investigated, three [chlordane (OR = 1.35; 95%CI 0.56–3.24), endosulfan (OR = 1.40; 95%CI 1.01–1.95), and heptachlor (OR = 1.13; 95%CI 0.46–2.80)] were positively associated with diabetes. This finding was consistent with a study which used US National Health and Examination Survey (NHANES) data that reported a strong association between diabetes and PCB, two dioxin congeners, and three organochlorine pesticides (oxychlordane, DDE, and trans-nonachlor) [10]. A study in the USA under the Agricultural Health Study program also found diabetes to be correlated with seven organochlorine pesticides (aldrin, chlordane, heptachlor, dichlorvos, trichlorfon, alachlor, and cyanazine) [14]. Compared with these previous results, ORs of chlordane in our study were higher (1.35 vs 1.16 in the previous study) but lower for heptachlor (1.13 vs 1.20). Our study is the first to show that endosulfan is strongly associated with diabetes. However, although endosulfan has long been banned in the Western world a long time ago, being one of the most toxic POPs, it was still widely used in Thailand until quite recently. In an animal study, exposure to endosulfan was shown to affect the endocrine cells of the pancreas of a rabbit and also that it decreased insulin secretion [27].

For organophosphates, in our study, specific compounds found to be positively associated with diabetes were chlorpyrifos (OR = 1.20; 95%CI 0.83–1.75), dicrotophos (OR = 2.12; 95%CI 0.97–4.61), dichlorvos (OR = 1.03; 95%CI 0.41–2.62), EPN (ethyl-p-nitrophenyl) (OR = 1.29; 95%CI 0.83–2.02), mevinphos (OR = 2.22; 95%CI 1.17–4.19), monocrotophos (OR = 1.29; 95%CI 0.72–2.32), and methamidophos (OR = 1.04; 95%CI 0.65–1.65). This is consistent with a study in the USA by Montgomery et al. [14], which reported an increased ORs of diabetes among subject exposure to chlorpyrifos (OR = 1.03; 95%CI 0.91–1.17), coumaphos (OR = 1.26; 95%CI 1.03–1.55), dichlorvos (OR = 1.21; 95%CI 0.98–1.49), phorate (OR = 1.22; 95%CI 1.06–1.42), terbufos (OR = 1.17; 95%CI 1.02–1.35), and trichlorfon (OR = 1.85; 95%CI 1.03–3.33). The ORs of chlorpyrifos and dichlorvos in our study were higher than those in the previous study. This issue will be discussed later. In an animal study, it was found that organophosphate inhibits acetylcholinesterase and causes an accumulation of acetylcholine and reduced insulin production [22, 28].

For carbamates, our results are in contrast with that reported by Montgomery et al. [14] in the USA. Of the four carbamate insecticides investigated (carbofuran/Furadan, carbosulfan, methomyl, and carbaryl/Sevin), we found that all were positively associated with diabetes. For carbaryl, the ORs were statistically significant (OR = 1.50; 95%CI 1.02–2.19). The OR in our study was higher than the results reported by Montgomery et al. [14] (OR = 1.10; 95%CI 0.95–1.28). In another animal study, carbaryl affected glucose homeostasis and insulin secretion [29].

We also found permethrin/Ambush, a pyrethoid insecticide, to be associated with diabetes (OR = 1.39; 95%CI 0.64–3.04). This finding has never been reported in an epidemiological study before. In molecular study, carbamate and pyrethoid compounds demonstrated a potential effect on circadian disruption which is linked to an increased diabetic risk [30].

For herbicides, of the 6 compounds investigated, four of them (butachlor, glyphosate, paraquat/gramoxone, and 2,4-D) could predict risk of diabetes but not statistically significant. A similar result was also reported in the Agricultural Health Study [14]. Evidence of the diabetic effect of herbicides was first recognized among Vietnam veterans who had used Agent Orange, which is an herbicide containing dioxins [31]. Dioxins were strongly associated with diabetes in [10]. A study in Australia compared the mortality of 1999 outdoor staff who had used insecticides in the period 1935–1996 to those of the general population [32] and that study reported an association between mortality as a result of diabetes and herbicide use, at the standard mortality rate (SMR) of 3.57 (95%CI 1.16–8.32). This is a high level of association.

Of the nine fungicides investigated, eight (benlate, carbendazim, copper sulfate, mancozeb, maneb, metalaxyl, thiophanate-methyl, and zineb) were positively associated with diabetes. For benlate, the association is statistically significant with OR of 2.08 (95%CI 1.03–4.20). Although associations between diabetes and fungicides have never been reported in any epidemiological study prior to this, a recent tissue-culture study reported that fungicide exposure can cause insulin resistance in fat cells and causes higher blood glucose [33].

Compared with the study in the USA [14], most of ORs found in our study were higher. The possible explanation for this is that Thai people are more likely to have poorer health backgrounds, life styles, and risky agricultural practices and behaviors. Data on PPE use and pesticide use behaviors that were also collected in this study showed that the rate of PPE utilized was lower than 30% and 41% of users did not change their clothes immediately after spraying the pesticides. A further localizing factor is that Thai people, particularly rural Thais, tend to eat less protein in their diet with greater amounts of vegetables and have poorer educational and socioeconomic status, all of which are confirmed diabetic risk factors [17].

The major sources of error in this type of study were recall and selection bias. Recall bias usually occurs when using past exposure data which may be biased due to differences in recall ability between case and control [34]. However, as information on pesticide and diabetic linkage was not well known in Thailand, the recall problem, if it did occur, would affect both case and control groups equally. As for selection bias, it often occurs when exposure or disease information are not accurate [35]. In our situation, the error for case selection was expected to be minimal since each case was a medically diagnosed diabetes patient. However, by using neighboring controls, it was very likely that the control subject was also exposed to some type of pesticides. This phenomenon would have the effect of lowering the degree of association between diabetes and any of the pesticides.

Data on exercise and the eating of various types of food, including vegetables, fruit, and sweet were also collected in this study for analysis (Table 1). However, since most of the study subjects were farmers who had similar lifestyles, exercise and eating factors failed to predict diabetic risk and therefore they were not included in the analytical model.

## Conclusion

Our results suggest that the occurrence of diabetes among Thai farmer was associated with pesticide exposure. The results supported the existing literature that pesticide exposure increases the risk of diabetes. Of the 35 individual pesticides investigated, those found to show statistically significant increased risk of diabetes were endosulfan, mevinphos, carbaryl/Sevin, and benlate. The ORs in this study were higher than previous studies in Western populations, and the difference is considered to be explainable by subject discrepancy in terms of health background, safety practices, and nutritional status. It must be noted that recall and selection bias tend to obscure the true association between diabetes and pesticides. It is suggested that more studies employing a larger sample size and better exposure tracking are still needed to confirm our results and to identify more individual compounds that may be a causal effect of diabetes. The issue should certainly receive more attention by public institutions, and practical measures to control those harmful pesticides are urgently needed.
